# *In Vivo* Disintegration and Bioresorption of a Nacre-Inspired Graphene-Silk Film Caused by the Foreign-Body Reaction

**DOI:** 10.1016/j.isci.2020.101155

**Published:** 2020-05-13

**Authors:** Linhao Li, Yanbing Liang, Guohang Wang, Peng Xu, Lingbing Yang, Sen Hou, Jin Zhou, Lizhen Wang, Xiaoming Li, Li Yang, Yubo Fan

**Affiliations:** 1 Key Laboratory for Biomechanics and Mechanobiology of Ministry of Education, School of Biological Science and Medical Engineering, Beihang University, Beijing 100191, China; 2 Beijing Advanced Innovation Center for Biomedical Engineering, Beihang University, Beijing 100191, China; 3 Key Laboratory of Biorheological Science and Technology of Ministry of Education, College of Bioengineering, Chongqing University, Chongqing 400030, China; 4 Beijing Key Laboratory of Rehabilitation Technical Aids for Old-Age Disability, National Research Center for Rehabilitation Technical Aids, Beijing 100176, China

**Keywords:** Biomimetics, Biomaterials, Natural Material

## Abstract

Graphene-based substrates are emerging as a promising functional platform for biomedical applications. Although dispersible graphene sheets have been demonstrated to be biodegradable, their assembled macroscopic architectures are biopersistent because of strong π-π interactions. In this study, we developed a nacre-inspired graphene-silk nanocomposite film by vacuum filtration with a subsequent green chemical reduction procedure. The “brick-and-mortar” architecture not only ensures the mechanical and electrical properties of the film but also endows it with disintegrable and bioresorbable properties following rat subcutaneous implantation. Furthermore, covalent cross-linking leads to the formation of graphene with decreased interlayer spacing, which effectively prolongs the residence time *in vivo*. We found that enzymatic treatment created microcracks on the film surface and that the foreign-body reaction was involved in the deformation, delamination, disintegration, and phagocytosis processes of the nanocomposite films. This bioinspired strategy paves the way for the development of high-performance graphene-based macroscopic biomaterials with tunable bioresorbability.

## Highlights

• Nacre-inspired graphene-silk nanocomposite film was developed by vacuum filtration
• The “brick-and-mortar” architecture ensures the mechanical and electrical properties
• The nanocomposite film has disintegrable and bioresorbable properties *in vivo*
• The foreign-body reaction was involved in the disintegration processes of the film

## Graphical Abstract

**Figure:**
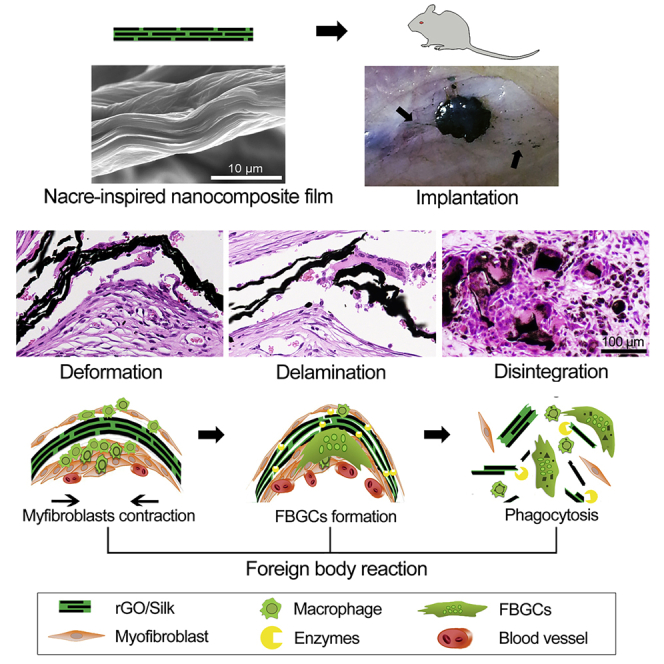

## Introduction

Bioresorbability/biodegradability is one of the most important considerations for the design and development of today's biomaterials. The term “biodegradation” refers to “a biological agents-mediated chemical process such as hydrolysis oxidative, and enzymatic mechanisms resulting in the breakdown of covalent bonds of materials.” The term “bioresorption” means that “the materials or their degradation products are removed by body fluid, tissues and cellular behaviors (e.g., phagocytosis) in a biological environment” (Treiser et al., 2013). Both definitions imply the temporary presence of functional implants in the body. It has been found in clinical practice that permanent implants often cause a variety of side effects, such as long-term immune rejection, chronic inflammation, and fibrosis induced by the mechanical mismatch at the tissue-implant interface, and necessitate secondary surgical removal after the failure of the implants (Holzapfel et al., 2013, Londono and Badylak, 2015, Ratner, 2015). Implants based on resorbable/degradable materials, which are mainly used as implantable drug release systems, guided tissue regeneration scaffolds, and resorbable electronic devices, can avoid the above disadvantages. At present, many permanent implant materials are still used clinically, and there are certain limitations on the selection of degradable materials. Alternative bioresorbable materials are mainly natural or synthetic polymers, which are inadequate to meet the requirements of various clinical applications, such as the lack of conductive materials with bioresorbability (Balint et al., 2014). Therefore, the design and development of new types of bioresorbable materials with excellent properties remain an important research challenge.

Graphene, as a two-dimensional carbonaceous material with extraordinary mechanical and electrical properties, is considered promising for biomedical applications such as implantable electronic devices and tissue engineering scaffolds (Geetha Bai et al., 2018, Kumar and Chatterjee, 2016, Reina et al., 2017). Most previous studies have been devoted to understanding the positive effects of graphene-based materials on physicochemical properties and cellular functions (Domínguez-Bajo et al., 2019, Lu et al., 2013, Lu et al., 2016). There is still some controversy about the bioresorbability/biodegradability of graphene-based biomaterials. Studies have found that nanoscale single-layer and few-layer pristine graphene sheets can be degraded by oxidative enzymes secreted by immune cells and can also be engulfed, decomposed, and absorbed by phagocytes (Kurapati et al., 2018, Li et al., 2019, Martín et al., 2019)^.^ However, graphene sheets can assemble into macroscopic architectures such as fibers, paper-like films, and three-dimensional monoliths (Cong et al., 2014), which are considered biopersistent (Kumar and Chatterjee, 2016, Guarnieri et al., 2018, Fadeel et al., 2018). Previous scientific evidence indicates that this behavior is mainly attributed to the dense interlayer spacing and hydrophobic π-π bonding, which make infiltration by water difficult and cause only very slow surface corrosion (e.g., as protective coatings) (Su et al., 2014, Zhu et al., 2010). Non-bioresorbability severely limits the usage of graphene in biomedical applications. Through the addition of graphene sheets to existing degradable biomaterial systems, biodegradability can be guaranteed (Li et al., 2012a, Ryan et al., 2018), but their mechanical and electrical properties are severely compromised. Unfortunately, owing to the inattention toward the interactions between components, as well as the disordered internal structures, it is difficult to balance the physical and biological benefits of most graphene-based composite materials together.

Nacre is a naturally forming brick-and-mortar-like structure, which is composed of brittle aragonite platelets (more than 95 vol%) connected by an organic component that serves as a glue or bridge between the platelets. The “brick-and-mortar” layered architecture with abundant interfacial interactions between inorganic platelets and organic protein endows nacre with outstanding mechanical properties (Tanay et al., 2008, Wegst et al., 2014). Graphene is an ideal candidate for use as a 2D building block for constructing artificial nacre nanocomposites. Small amounts of organic components are evenly distributed between graphene sheets and are stably chemical bonded. The unique hierarchical structure and synergistic effects of interfacial interactions not only can greatly improve the overall mechanical performance but also maintain the electrical properties of graphene (Wan et al., 2018a, Wan et al., 2018b, Zhang et al., 2016). Importantly, the organic components increase the interlayer spacing of graphene and reduce the formation of π-π bonding interactions, which may lead to disintegration *in vivo* (Hung et al., 2014, Li et al., 2012b). Therefore, we hypothesize that the use of biodegradable organic components between graphene layers may lead to the disintegration of the whole nacre-biomimetic graphene-based nanocomposites, which will eventually be biodegraded. To test this hypothesis, we believe that organic components should have the following characteristics: (1) biodegradability; (2) excellent biocompatibility; (3) tunable chemical bonding with graphene; (4) a certain structure and mechanical retention in physiological environments due to the low content of the organic component in the composite system.

In this study, we selected silk fibroin (SF) as an organic component in graphene-based nanocomposites. SF, as a natural protein, has excellent biocompatibility, controllable biodegradability, long-term property retention *in vivo*, and high mechanical performance (Altman et al., 2003, Kundu et al., 2013). Importantly, it is possible to design a dense network of synergistic interfacial interactions between graphene oxide (GO) sheets and silk protein molecules, including hydrogen bonding, hydrophobic-hydrophobic interactions, and amide covalent bonds (Grant et al., 2016, Hu et al., 2013a, Hu et al., 2013b, Ling et al., 2014). On the other hand, there are different macroscopic architectures of nacre-biomimetic structural materials (e.g., fiber [Ma et al., 2018], film [Wang et al., 2018], and 3D bulk [Gao et al., 2017, Yang et al., 2019]). In this work, we used a macroscopic graphene-based material with a simple film architecture for experimental verification. GO/silk biomimetic nanocomposite films were fabricated by vacuum filtration. The interfacial bonding between GO and silk was controlled by whether (or not) the nanocomposites were subjected to covalent cross-linking. Then, we used a biosafe reducing agent, vitamin C (VC), to reduce the films. In addition to the analysis of the mechanical and electrical properties of the nanocomposite films, we carried out subcutaneous implantation in rats to verify the disintegration and bioresorption of the films *in vivo*. Finally, the mechanisms of foreign-body reactions (FBRs) that contributed to their disintegration were explored. Our results demonstrate the successful construction of an *in vivo* disintegrable and bioresorbable graphene-based material using a nacre-biomimetic structure.

## Results

### Fabrication and Characterization of Nacre-Inspired Graphene/Silk Nanocomposite Films

**Figure 1:**
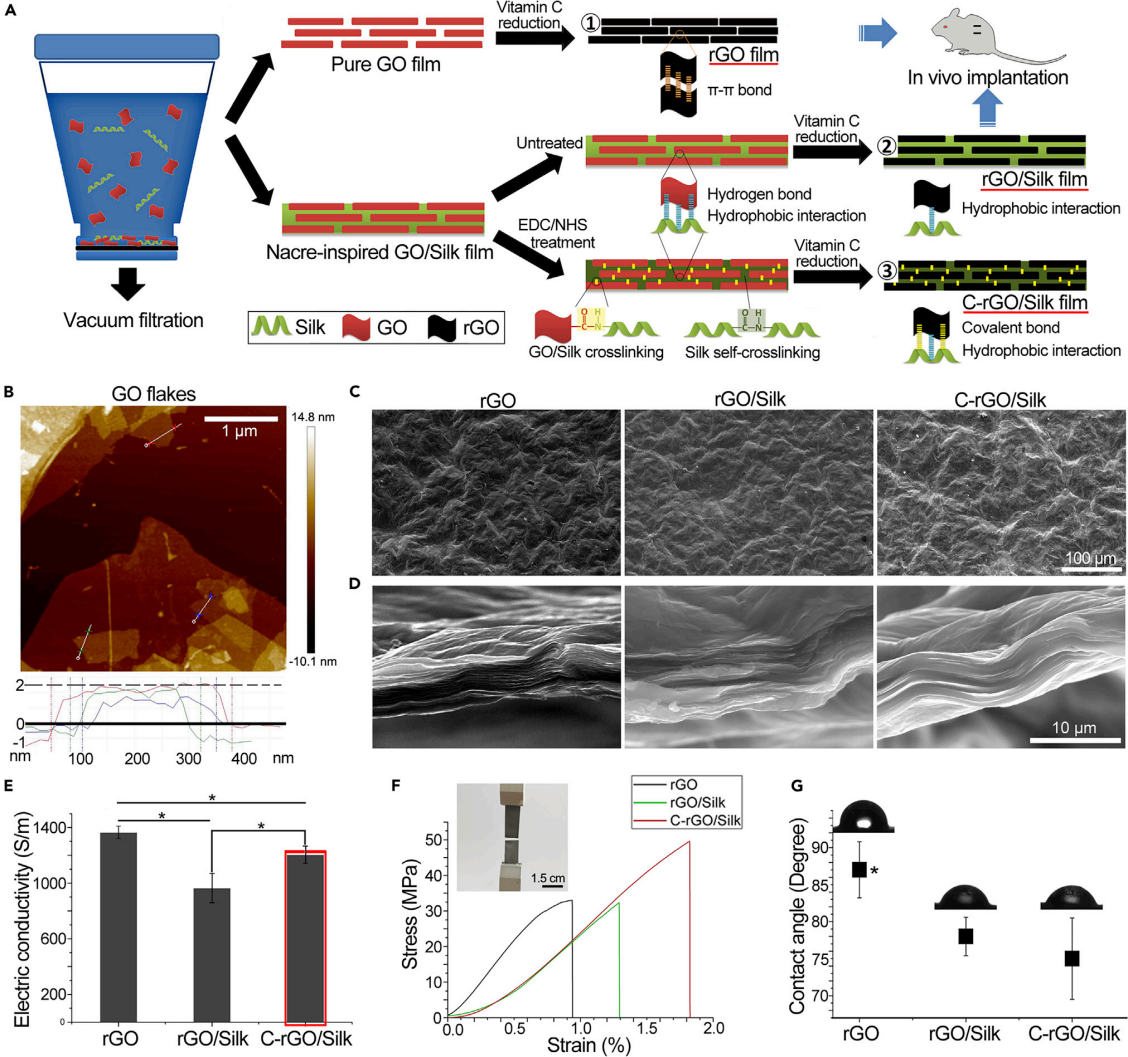
Fabrication and Characterization of Nacre-Inspired Graphene/Silk Nanocomposite Films (A) Schematic illustration of the fabrication of rGO/silk (2.5% w/w silk content) nanocomposite films by vacuum filtration with and without cross-linking. ① rGO; ② rGO/silk (non-cross-linked); ③ C-rGO/silk (covalently cross-linked). (B) AFM image of GO flakes and corresponding height profiles. (C) SEM images of the surface view of rGO, rGO/silk, and C-rGO/silk films. (D) SEM images of the cross-sectional view of rGO, rGO/silk, and C-rGO/silk films. (E) Electrical conductivity of rGO, rGO/silk, and C-rGO/silk films. ∗p < 0.05. Data are presented as the mean ± SD (n = 4). (F) Mechanical properties of rGO, rGO/silk, and C-rGO/silk films. (G) Water contact angles of rGO, rGO/silk, and C-rGO/silk films. ∗p < 0.05. Data are presented as the mean ± SD (n = 4).

The process for fabricating freestanding reduced graphene oxide (rGO), rGO/silk, and cross-linked rGO (C-rGO)/silk films is illustrated in Figure 1A. In brief, an SF solution was added dropwise to a GO suspension under continuous stirring. The pH of the mixed solution was adjusted to 10 to form a homogeneous solution, and the final mass ratios of graphene to silk were 95:5 and 97.5:2.5. Few-layer GO flakes with a size and thickness of 0.5–1 μm and 0.75–2 nm, respectively, were characterized by atomic force microscopy (AFM) (Figure 1B). Then, the GO/silk mixture solution was filtered by vacuum-assisted filtration and assembled into a GO/silk nanocomposite film. For the cross-linked GO (C-GO)/silk film, the film was soaked successively in 1-ethyl-3-carbodiimide hydrochloride (EDC)/N-hydroxysuccinimide (NHS) solution, thereby providing a water-stable C-GO/silk film in which the silk molecules were bonded with adjacent GO flakes (GO-silk) and themselves (silk-silk) through covalent amide linkages. Finally, the GO, GO/silk, and C-GO/silk films were reduced by VC to recover the π-π conjugated structure of the graphitic lattice. SEM images showed that there were no obvious differences in surface morphology among the rGO, rGO/silk, and C-rGO/silk films (Figure 1C). The cross-sectional views of the rGO and rGO/silk films show a nacre-like layered structure with rough morphology. In the C-rGO/silk film, the interface between layers is not clear, and a fusion phenomenon appears (Figure 1D). This may be due to the covalent bonding between the organic and inorganic phases and the silk self-cross-linking, which reduces the interlayer spacing. In this work, electric conductivity is the most important consideration for the selection of silk content in the nanocomposite films. Among the three groups, pure rGO films exhibit the highest conductivity (1,366 ± 43.5 S/m). Compared with the conductivity of the pure rGO films, the conductivity of nanocomposites containing 2.5% silk decreased to 965 ± 105.6 S/m, whereas that of nanocomposites with 5% silk decreased approximately 4-fold (365 ± 55.8 S/m) (Figure S1A). Therefore, in the subsequent experiments, we used a 2.5% silk nanocomposite film. Importantly, cross-linking can significantly improve the electrical conductivity to 1,205 ± 62.5 S/m (Figures 1E and S1B). We further investigated the mechanical properties of different films, such as the stress-strain curves, tensile strength, and elongation at break (Figures 1F and S2). The rGO films initially showed hard and brittle character, with a high Young's modulus (4.12 ± 0.48 GPa) and an elongation at break of 0.98 ± 0.243%. With the addition of silk, the modulus of the graphene films decreased, but their elongation at break increased to 1.26%. For the C-rGO/silk film, the ultimate tensile strength increased to 52.5 ± 4.8 MPa, significantly higher than that of the other films. Furthermore, the C-rGO/silk film retained a relatively high elongation at break (1.82 ± 0.26%). We also found that the addition of silk can increase the hydrophilicity of the nanocomposite film, both of non-cross-linked and cross-linked groups (Figure 1G).

### Effect of Cross-Linking on Structural Retention of Graphene/Silk Nanocomposite Films

**Figure 2:**
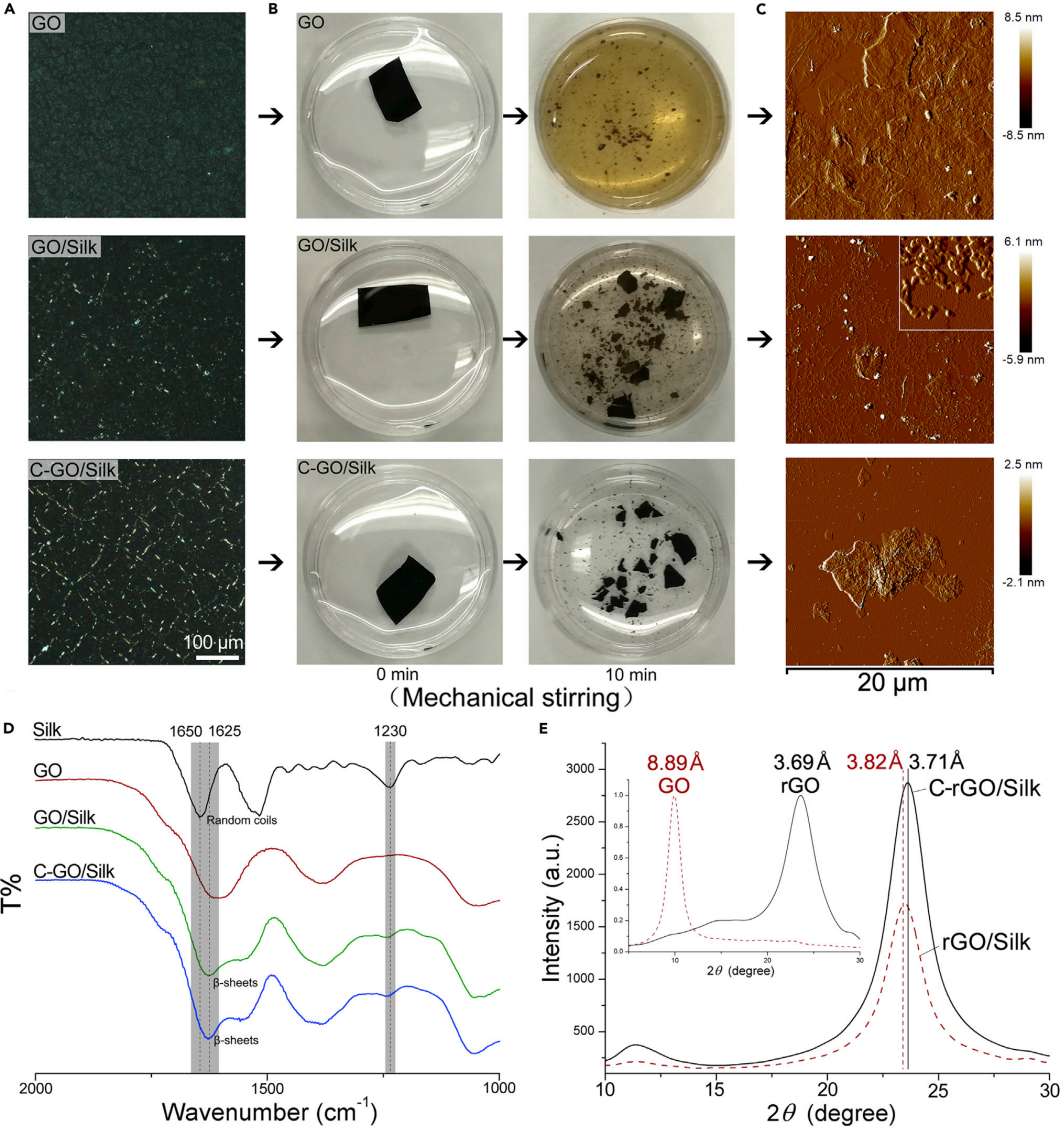
Effect of Cross-Linking on Structural Retention of Graphene/Silk Nanocomposite Films (A) Polarized microscopy images of GO, GO/silk, and C-GO/silk films. Covalent cross-linking changes the surface microstructure of the composite films. (B) Photographs of GO, GO/silk, C-GO/silk films after being soaked and stirred in water for 0 and 10 min with a magnetic stirrer at the same rotational speed. (C) AFM images of suspensions of different films stirred by magnetic force for 10 min. (D) FTIR analysis of SF powder and GO, GO/silk, and C-GO/silk films. The interactions of SF with GO mediate the formation of SF β sheet structures. (E) Powder XRD patterns of GO, rGO, rGO/silk, and C-rGO/silk films. The d-spacing of the rGO/silk film shifted from 3.82 to 3.71 Å after cross-linking. Inset: Powder XRD patterns of GO and rGO films. The d-spacing of GO shifted from 8.89 to 3.69 Å after reduction.

To further analyze the interaction between silk and GO and the effect of covalent cross-linking on the properties of the films, we examined the physicochemical properties of the nanocomposite films before VC reduction. Polarization microscopy can effectively identify different surface microstructures of the films (Figure 2A). Compared with the pure GO film, the GO/silk film exhibited nonhomogeneous crystal-like spots on its surface. This phenomenon may be caused by the ordered β sheet structure of silk molecules. After covalent cross-linking, the film showed interconnected network-like bright spots. We believe that the formation of stable covalent bonds between GO and silk and between silk and silk makes the film components more closely connected, increasing the ordering of the internal molecular arrangement. Moreover, we observed that the nanocomposite films have different structural retention properties in water by mechanical stirring before reduction. After stirring for 10 min, the GO film was quickly dispersed in water and showed a light-yellow suspension. Compared with the pure GO film, the GO film to which SF was added was significantly resistant to disintegration in water at the same time point. Especially after cross-linking, although there were large fragments, the suspension was still relatively clear (Figures 2B and S3). We performed AFM analysis of the suspensions, and similar dispersibility results were observed. Furthermore, free silk nanofilaments were found in the non-cross-linked nanocomposite film suspension. By contrast, in the cross-linked samples, silk nanofilaments were found on the surface of the GO flakes (Figures 2C and S4). These results show that EDC/NHS covalent cross-linking can effectively enhance the interaction between SF and GO. To analyze conformational changes of the silk incorporated into the GO films, we characterized lyophilized silk powder, GO film, GO/silk film, and C-GO/silk film samples using Fourier transform infrared (FTIR) spectroscopy (Figure 2D). Compared with the pure GO film, the GO/silk film exhibited additional characteristic peaks at 1,625 cm^−-1^ (amide I; carbonyl stretching) and 1,230 cm^−1^ (amide III; C‒N stretching and N‒H deformation). Compared with the corresponding peak of the lyophilized SF powder, the amide I peak of the GO/silk film shifted from 1,650 to 1,625 cm^−1^, indicating the presence of stabilizing β sheet structures after the interaction of SF molecules with GO. The formation of these β sheets was induced by fluid shear force during vacuum filtration and rearrangement of SF molecules during drying (lowest energy conformation, β sheets), thus providing noncovalent physical cross-links between the GO and silk molecules (hydrophobic interactions). After covalent cross-linking, the secondary structure of silk remained unchanged. X-ray diffraction (XRD) analysis of cross-linked and non-cross-linked GO/silk films revealed that the C-rGO/silk film has a stronger graphite peak than the rGO/silk film, indicating a higher degree of orientation and crystallinity (Figure 2E). We also found that the d-space decreased from 3.82 to 3.71 Å after cross-linking, which indicated that covalent cross-linking can reduce the interlayer spacing of graphene and make the internal structure denser. A denser layer spacing may lead to the cross-linked nanocomposite film having high electrical conductivity and mechanical properties.

### *In Vivo* Deformation, Delamination, and Disintegration of Different Films

**Figure 3:**
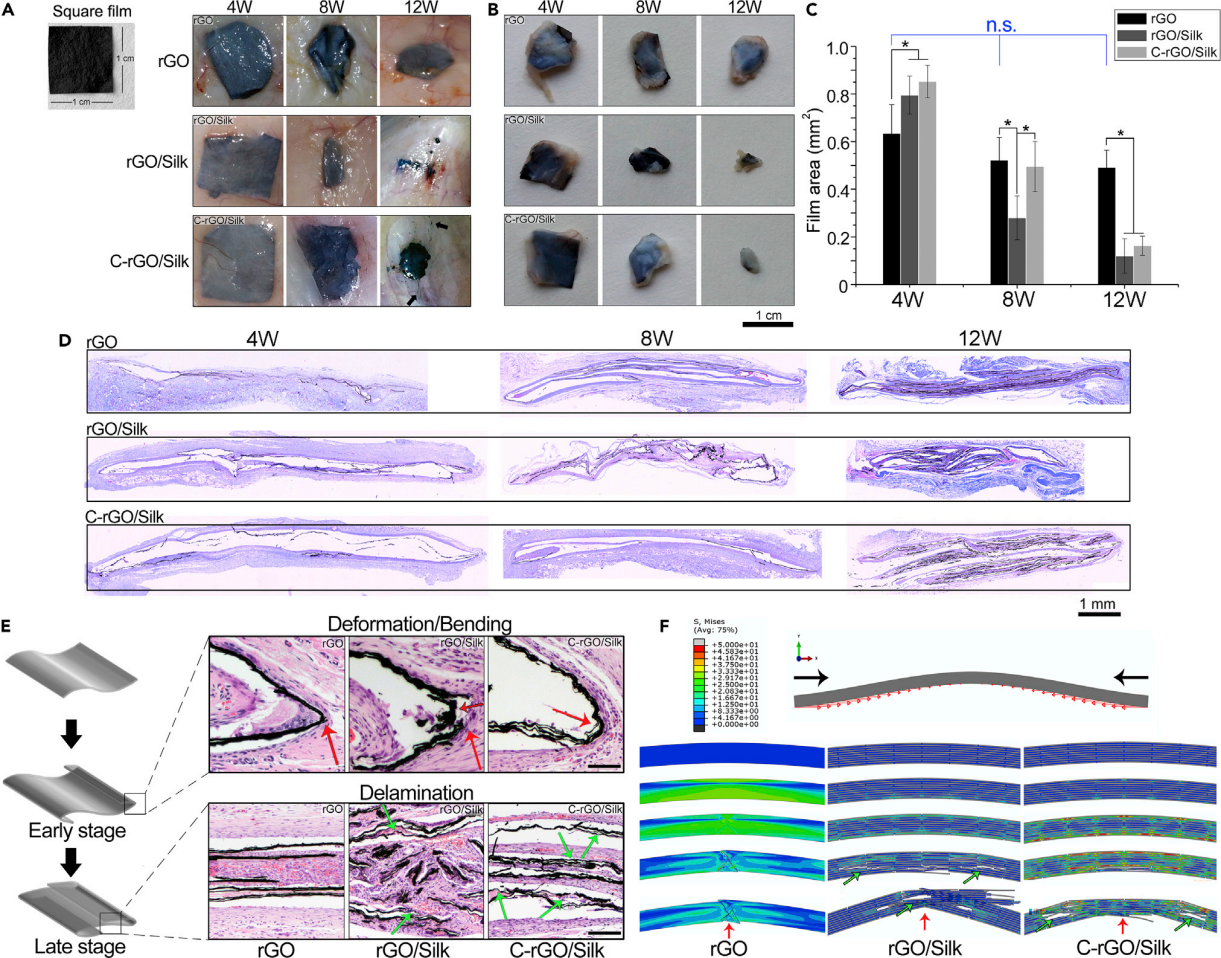
*In Vivo* Deformation, Delamination, and Disintegration of Different Films (A) Photographs of rGO, rGO/silk, and C-rGO/silk films *in situ* of the subcutaneous tissue at 4, 8, and 12 weeks after implantation. The black arrows indicate the graphene debris dispersed in the tissue. (B) Photographs of rGO, rGO/silk, and C-rGO/silk films removal from subcutaneous tissue at 4, 8, and 12 weeks after implantation. (C) Quantitative analysis of the changes in the areas of different films at different times after implantation. ∗p < 0.05. Data are presented as the mean ± SD (n = 3). “n.s.” means no significance. (D) Representative whole-slide images of Masson's trichrome staining of histological sections of different films at 4, 8, and 12 weeks after subcutaneous implantation. (E) Schematic illustration of the degree of deformation and delamination of the film at different stages (left panel), indicating the positions of different films shown in the H&E staining images (right panel) of histological sections of different films at 8 and 12 weeks after implantation. Red arrows indicate the bent edge regions, and green arrows indicate the internal delaminated regions of different films. (F) FE simulation analysis of the deformation of different films under external force. The films have different bending and delamination behavior due to different internal structures and interfacial interactions between components under continuous peripheral shear stress. Red arrows indicate the positions of bending, and green arrows indicate delamination.

In this study, we used a rat subcutaneous implantation model to evaluate the *in vivo* biological response to the nanocomposite films at different stages. Before the *in vivo* implantation, we performed live/dead cell viability assay on different nanocomposite films, and the results did not reveal that these films were cytotoxic (Figure S5). The photographic results showed that, at 4 weeks after initial implantation, the rGO film had a high degree of deformation and the overall area considerably decreased (Figures 3A and S6A). By contrast, the rGO/silk and C-rGO/silk samples mainly retained their original appearance. At 8 weeks after implantation, the square films all underwent different degrees of deformation and shrinkage. Compared with the rGO and C-rGO/silk films, the rGO/silk film showed significant shrinkage. In the late stage of implantation (12 weeks), the apparent morphology of the rGO/silk film showed the most obvious changes among the three films. There was small scattered graphene debris within the subcutaneous tissue, and some samples were even difficult to find (Figure 3A, middle panel). The area of the C-rGO/silk film also decreased considerably over time, and small scattered black debris appeared around the sample (Figure 3A, black arrows in the lower panel). However, the apparent morphology of the rGO film did not change significantly, and no breaking or disintegration occurred (Figures 3A and 3B, upper panel). We also used ImageJ software to analyze the area of the film samples after removal (Figure 3C). As the implantation time prolonged, we found no significant decrease in the size of the rGO samples. However, the area of the rGO/silk films decreased sharply and the area of the C-rGO/silk films also showed a relatively linear downward trend.

We further observed changes in the structure of the different films in tissue sections (Figure 3D). At the early stage of implantation (4 weeks), the rGO film underwent overall deformation and bending at the edges, whereas the nanocomposite films retained a relatively complete structure. This result indicated that improvement in the mechanical strength of the nacre structure can help to maintain the structural stability after the early implantation stage (Figure 3D, left panel). With the extension of the implantation time (8 weeks), many crimp structures appeared in rGO films, but this state did not change further after 12 weeks. However, in the late stage of implantation, the non-cross-linked and cross-linked nanocomposites exhibited not only bending and curling but also delamination and disintegration. Notably, the C-rGO/silk films still retained a relatively complete structure 8 weeks after implantation, whereas the rGO/silk films had considerable fracture and delamination, indicating that the cross-linked films have better structural stability *in vivo* (Figure 3D, middle panel).

Because edge bending occurs during the early implantation stage, which may be the key phenomenon in the delamination and disintegration behaviors of the films *in vivo*, we used H&E staining to observe the cell distribution at the bent edge of the film (Figure S6B). The results showed that numerous host cells appeared at the bent edge (inside the film), indicating that the host response *in vivo* may be involved in the deformation process. We describe the deformation process of the rGO film *in vivo* through cartoon pictures in Figure S6C: the rGO film is initially square, the edge of the film bends, and the whole film curls.

We further investigated the relationship between the structure and function of different nanocomposite films. At 8 weeks after implantation, we observed that the bending regions of the rGO, rGO/silk, and C-rGO/silk films have different morphological characteristics. The rGO film has a sharp corner at the bending part, but the rGO/silk and C-rGO/silk films have a certain curvature (Figure 3E, red arrows). We also observed partial cracking in the rGO/silk films. At 12 weeks after implantation, we observed that, in regions inside the overall crimped films, the SF-added nanocomposite films (rGO/silk and C-rGO/silk films) showed delamination, whereas the rGO film remained relatively intact. In the rGO/silk sample, a large amount of debris was dispersed in the surrounding tissue owing to the concurrence of delamination and fracture (Figure 3E, green arrows). To explain the bending and delamination characteristics of different films, we carried out a finite element (FE) simulation analysis (Figure 3F). From the simulation results, it can be found that, under the action of continuous external force, the nanocomposite films have similar delamination and fracture behavior, which showed that the different bonds between the graphene and silk layers are the key factors governing their bending and delamination characteristics. However, regarding the mechanical properties, morphological characteristics, and structure-function relationships of the films, the following two questions are still difficult to answer: (1) Why is there constant external contraction? (2) Why do the nanocomposite films with good mechanical properties still undergo deformation and disintegration at a later stage?

### Foreign-Body Reaction to Different Films

**Figure 4:**
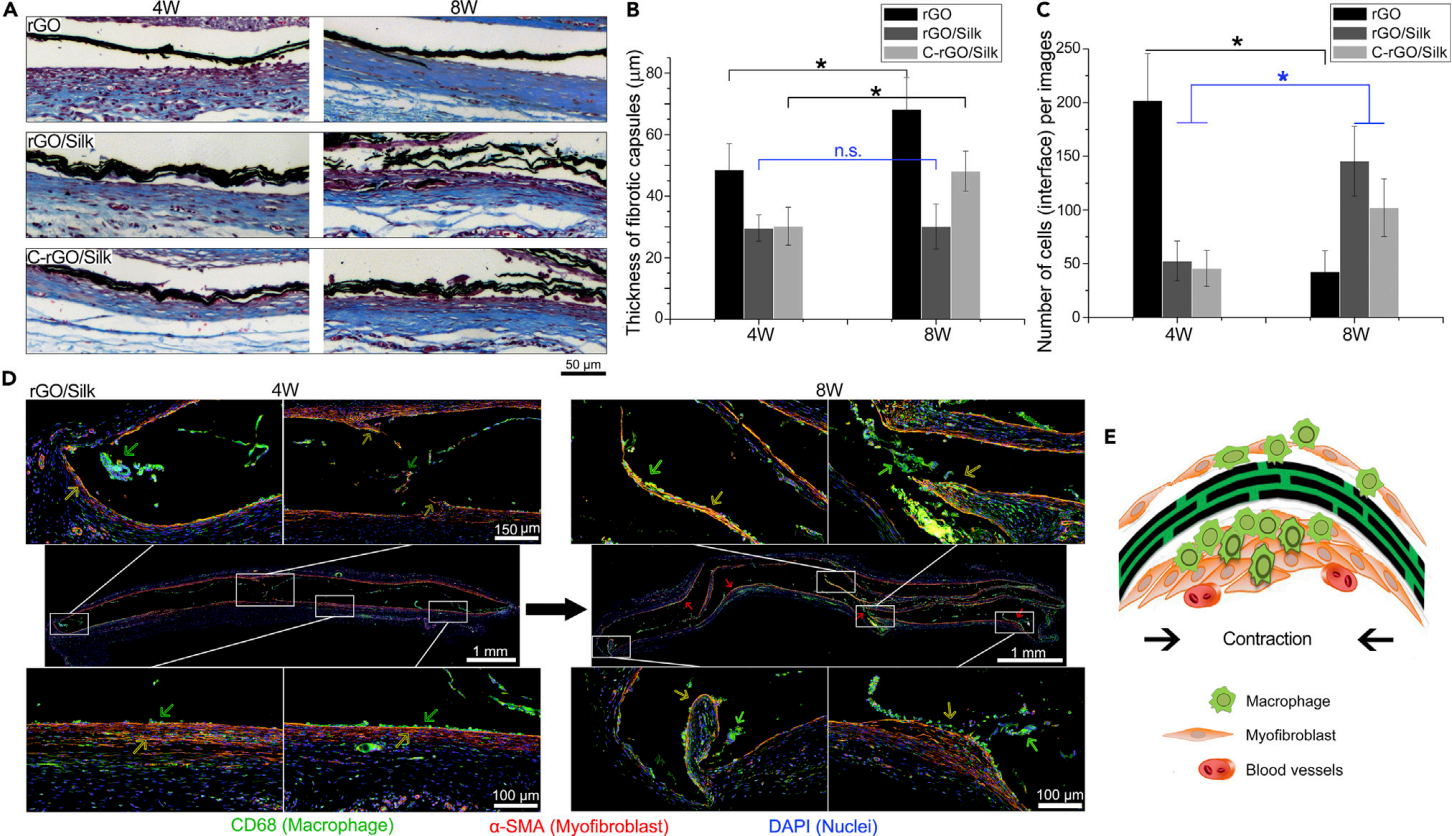
Foreign-Body Reaction to Different Films (A) Representative images of Masson's trichrome staining of histological sections of different films at 4 and 8 weeks after subcutaneous implantation. (B) Statistical analysis of the thickness of the fibrotic capsules surrounding the different films at 4 and 8 weeks after implantation. ∗p < 0.05. Data are presented as the mean ± SD (n = 6). “n.s.” means no significance. (C) Statistical analysis of the number of host cells at the tissue/film interface. ∗p < 0.05. Data are presented as the mean ± SD (n = 8). (D) Representative whole-slide image of immunohistofluorescence staining for CD68, α-SMA, and DAPI of histological sections of rGO/silk films at 4 and 8 weeks after implantation. The images show that the macrophages and myofibroblasts aggregate and infiltrate at the bending region of a curved film over time. (E) Schematic illustration of the aggregation and infiltration of macrophages and myofibroblasts at the bending region of a curved film in the FBR process reaction, indicating that the contraction of myofibroblasts may cause film deformation.

To answer the above questions, we further evaluated the biological response/FBR to the nanocomposite films *in vivo*. Masson's trichrome staining can reflect the thickness of the collagenous fibrous capsules formed around the films at different time points (Figure 4A). At 4 weeks after implantation, fibrous capsules appeared on the surface of all three types of films, among which the rGO films had the thickest fibrous capsules and the C-rGO/silk films had the thinnest. At 8 weeks after implantation, the thickness of the fibrous capsule on the surface of the rGO film was significantly increased, whereas only a small number of host cells remained. These results indicate that the FBR induced by rGO film implantation has tended to enter the end stage (quiescent stage). This phenomenon is similar to the FBR caused by bioinert materials such as silica-based implants. By contrast, the thickness of the fibrous capsule on the surface of the non-cross-linked rGO/silk film remained basically unchanged; however, the number of host cells was significantly increased. Similarly, the number of host cells surrounding the C-rGO/silk films also increased (Figures 4B and 4C). This finding indicates that continuous changes in the structure or properties of implanted materials also trigger different responses of host cells during the FBR. According to the tissue section results, changes in the structure of the nanocomposite films such as bending and delamination may be closely related to the phenotypes and distribution of the host cells. Therefore, we analyzed the phenotypes and distribution of the host cells at 4 and 8 weeks after implantation of the rGO/silk film (Figure 4D). Immunofluorescence images showed that there were more CD68^+^ macrophages at the bending or delaminated region of the film than elsewhere in the film and that the macrophages tended infiltrate into the interior of the films over time. Meanwhile, we found that the macrophages at the bending region of the film were also accompanied by numerous α-SMA^+^ myofibroblasts. We conclude that macrophages aggregate in the depressions and wrinkles in the film in the early stage and recruit or mediate the differentiation of myofibroblasts in the later stage (Figure 4E). The aggregation of contractile myofibroblasts can continuously affect the bending and deformation of thin films, which may be the main source of the sustained external forces on the films (Hinz, 2010).

### Aggregation and Distribution of Macrophages and Myofibroblasts in Different Films

**Figure 5:**
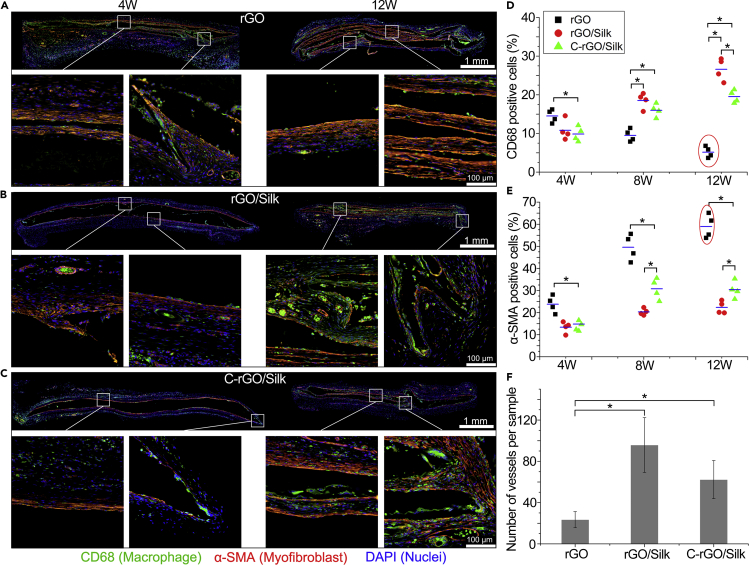
Aggregation and Distribution of Macrophages and Myofibroblasts in Different Films (A) Representative whole-slide images of immunohistofluorescence staining for CD68, α-SMA, and DAPI of histological sections of rGO film at 4 and 12 weeks after implantation. (B) Representative whole-slide images of immunohistofluorescence staining for CD68, α-SMA, and DAPI of histological sections of rGO/Silk film at 4 and 12 weeks after implantation. (C) Representative whole-slide images of immunohistofluorescence staining for CD68, α-SMA, and DAPI of histological sections of C-rGO/Silk film at 4 and 12 weeks after implantation. (D) Statistical analysis of CD68^+^ (macrophage) cell percentages surrounding different films at 4, 8, and 12 weeks after implantation. ∗p < 0.05. Data are presented as the mean ± SD (n = 4). (E) Statistical analysis of α-SMA^+^ (myofibroblast) cell percentages surrounding different films at 4, 8, and 12 weeks after implantation. ∗p < 0.05. Data are presented as the mean ± SD (n = 4). (F) Statistical analysis of the number of blood vessels surrounding the different films at 12 weeks after implantation. ∗p < 0.05. Data are presented as the mean ± SD (n = 6).

We further analyzed the aggregation and distribution of macrophages and myofibroblasts at the material/tissue interface at the early (4 weeks) and late stages (12 weeks) of implantation by immunofluorescence staining (Figure 5). The rGO film showed edge bending at 4 weeks after implantation (Figure 5A). The macrophages were accompanied by myofibroblasts mainly gathering at the bending region and infiltrating into the film. From a distribution point of view, the macrophages appeared in front of the myofibroblasts and may have a guiding effect on myofibroblast growth and migration. At 12 weeks after implantation, the number of macrophages both at the edge and inside the bending regions decreased significantly, and the number of myofibroblasts increased significantly. Similar to the Masson's trichrome staining results, these results indicate that the FBR induced by rGO film implantation entered the end stage. By contrast, the rGO/silk and C-rGO/silk films retained relatively complete film morphology at 4 weeks after implantation (Figures 5B and 5C). The aggregation of fewer macrophages and myofibroblasts indicates that the addition of silk protein can reduce the initial FBR. At 12 weeks after implantation, there was only a small increase in the number of myofibroblasts, whereas the number of macrophages increased significantly, especially in the non-cross-linked rGO/silk group (Figures 5D and 5E). We also found that the trends in the number of blood vessels and macrophages at the tissue/film interface and in the infiltrated tissue regions were similar, with the highest number in the rGO/silk group and the lowest in the rGO group (Figure 5F). These results showed that, in the rGO group, the films induce a strong FBR and the cells tend to be the quiescent phase at the late stage of implantation, forming a thick fibrous capsule; the nanocomposite films induce a lower FBR than the rGO films in the early stage, but a large number of macrophages appeared in the later stage, which indicates that the appearance of graphene debris can reactivate the host phagocytes.

### The Formation of Microcracks in Graphene/Silk Films by Enzymatic Treatment

**Figure 6:**
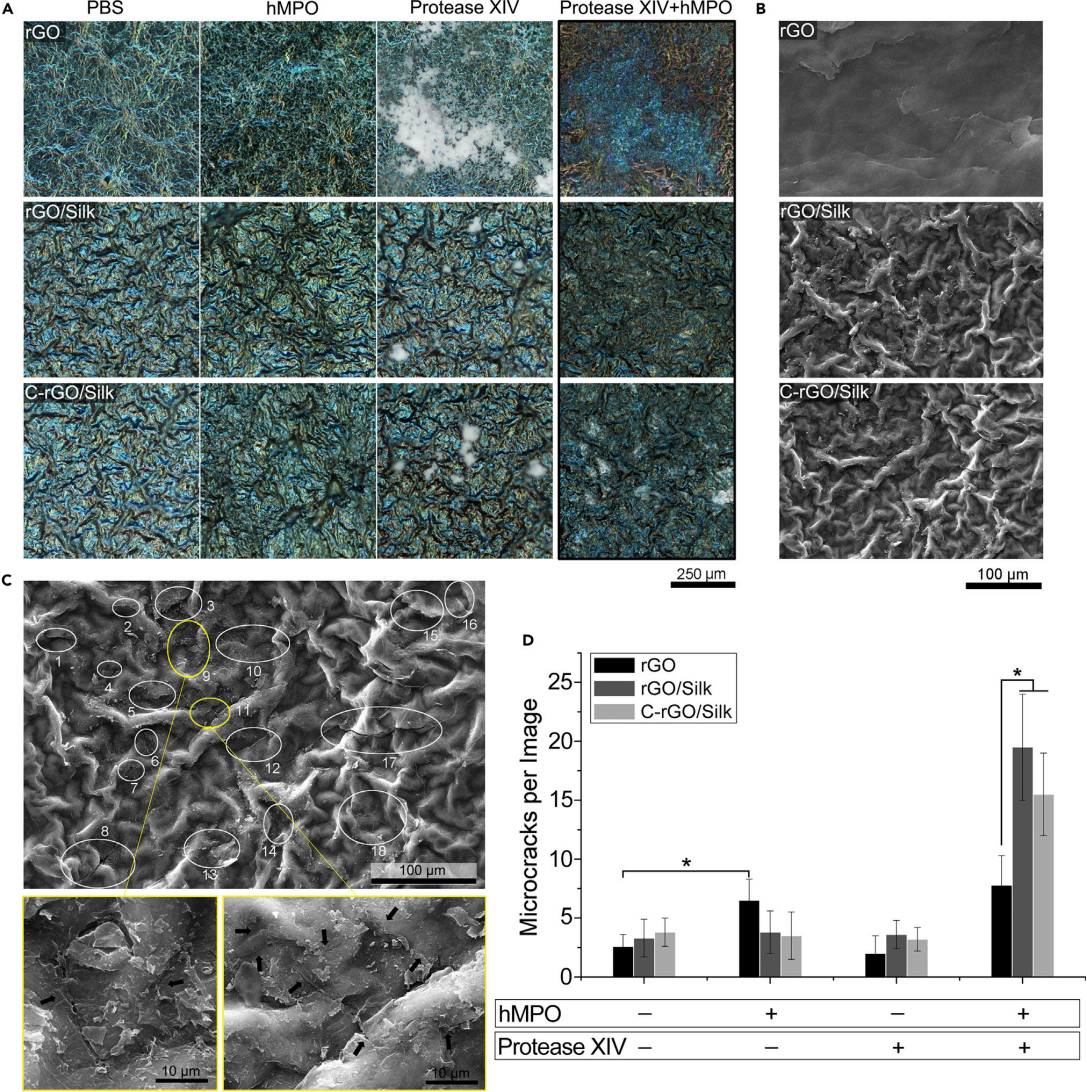
The Formation of Microcracks in Graphene/Silk Films by Enzymatic Treatment (A) Polarized microscopy images of rGO, rGO/silk, and C-rGO/silk films after treatment with PBS, hMPO, protease XIV, or hMPO/protease XIV in succession for 48 h. (B) SEM images of the different films after treatment with hMPO/protease XIV in succession for 48 h. (C) High-magnification SEM analysis of the formation of microcracks in rGO/silk films after treatment with hMPO/protease XIV in succession for 48 h. White circles and numbers indicate the location and number of microcracks marked in a single SEM image. The images in the yellow box below is the high-magnification images. (D) Statistical analysis of the numbers of microcracks on the surfaces of different films after treatment with PBS, hMPO, protease XIV, or hMPO/protease XIV in succession for 48 h. ∗p < 0.05. Data are presented as the mean ± SD (n = 8).

The nanocomposite films showed a large amount of dispersed debris in the late stage of implantation, and we believe that this debris may have an important correlation with the enzymes secreted by the immune cells involved in the FBR. Neutrophils, macrophages, and foreign-body giant cells (FBGCs) are capable of secreting large amounts of proteases and peroxidases to degrade biomaterials. Peroxidases also have a strong oxidative degradation effect on carbon-based materials (Kagan et al., 2010). It has been reported that human myeloperoxidase (hMPO) can effectively degrade nanoscale single-layer and multilayer graphene sheets (Kurapati et al., 2018). Furthermore, proteases can degrade natural proteins such as silk (Thurber et al., 2015). Therefore, we simulated the effects of peroxidase and protease treatment on graphene-based films *in vitro* (Figure 6). Polarization microscopy can detect the optical properties of the material surface and analyze the fine surface structure changes. According to the polarized microscopy results, wrinkles and color changes occurred on the surface of the three films after hMPO treatment and protease had no obvious effect on the films except for being strongly adsorbed on their surfaces. However, double enzyme treatment had a significant effect on the surface morphology of the different films (Figure 6A). SEM images showed that microcracks appeared on the surface of all the films after double enzyme treatment (Figure 6B). Among the three types of films, the largest number of microcrack structures was found in the non-cross-linked rGO/silk film samples (Figures 6C and 6D). The results of *in vitro* simulation experiments showed that enzymatic treatment can effectively affect the surface microstructure of graphene-based films. The emergence of many microcracks may play a key role in the overall disintegration of the films.

### Phagocytosis of Graphene/Silk Films at the Late Stage of Implantation

**Figure 7:**
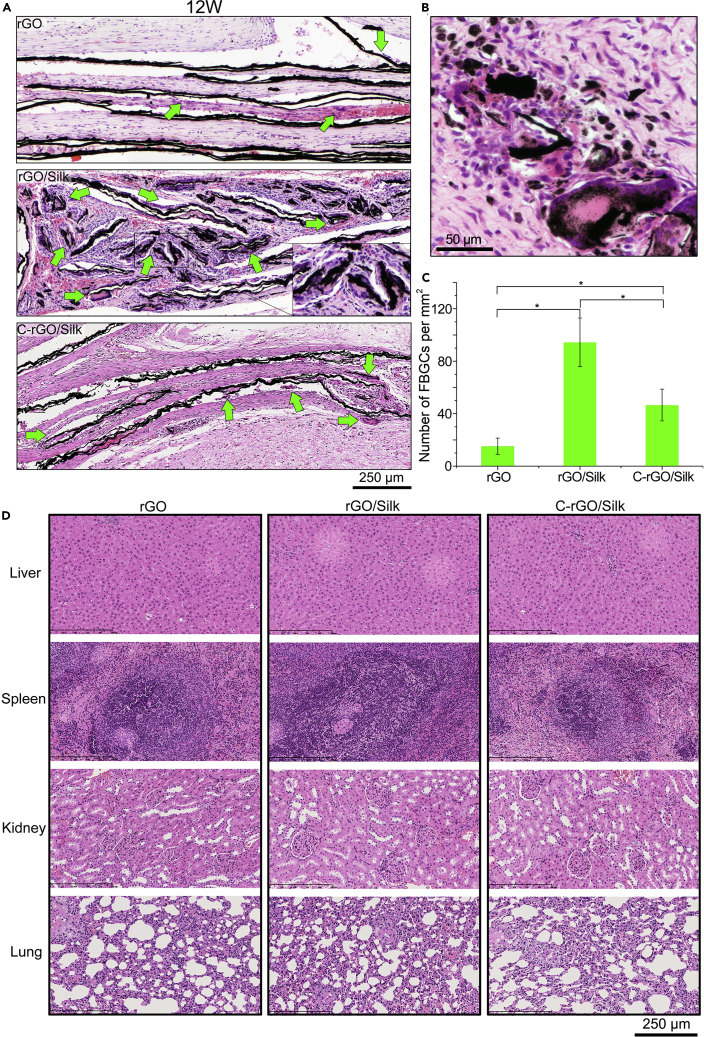
Phagocytosis of Graphene/Silk Films at the Late Stage of Implantation (A) Representative images of the H&E staining of histological sections of rGO, rGO/silk, and C-rGO/silk films at 12 weeks after implantation. The images show that the different films can induce different numbers of FBGCs after implantation. Green arrows indicate FBGCs. (B) Representative high-magnification images of the H&E staining of histological sections of rGO/silk films after 12 weeks of implantation. The images show the phagocytosis of FBGCs in the late stage of the FBR. (C) Statistical analysis of the number of FBGCs surrounding different films at 12 weeks after implantation. ∗p < 0.05. Data are presented as the mean ± SD (n = 4). (D) Representative images of the H&E staining of histological sections of the liver, spleen, kidney, and lung at 12 weeks after implantation of the different films. The images show no obvious accumulation of graphene debris.

At the late stage of the FBR, multiple macrophages can fuse to form FBGCs. FBGCs can secrete large amounts of oxidases and proteases to further decompose the implanted materials accompanied by phagocytosis (Al-Maawi et al., 2017). The formation of FBGCs and the occurrence of phagocytosis were observed by H&E and Masson's trichrome staining (Figures 7A and S7). Other than the overall bending deformation, the local structure of the rGO film was relatively complete, and there was no obvious fracture, delamination, or disintegration. At the same time, only a small number of FBGCs appear at the interface between the tissue and rGO film. By contrast, the rGO/silk films produced abundant black debris in the tissue and induced the appearance of the largest number of FBGCs among the three types of films. In the local structure of the C-rGO/silk films, there was obvious fracture and delamination, and the number of FBGCs was significantly higher than that surrounding the rGO films and lower than that surrounding the rGO/silk films (Figures 7A and 7C). High-resolution H&E staining images showed that the rGO/silk and C-rGO/silk nanocomposite films caused the appearance of abundant small scattered black graphene debris inside the FBGCs, indicating that phagocytosis occurred (Figure 7B). In addition, we tested the main organs (liver, spleen, kidney, and lung) of rats and found no accumulation or residue of black graphene debris (Figure 7D). In summary, the nacre-inspired nanocomposite films maintained a stable overall structure in the early stage of implantation but could be disintegrated and engulfed in the later stage. Furthermore, covalent cross-linking can significantly prolong the structure retention *in vivo*, so the process can be tuned by different bonds between the graphene flakes and silk protein molecules.

## Discussion

This study mainly focuses on the design of graphene-based artificial nacre nanocomposites for constructing high-performance implantable biomaterials. These nanocomposites not only provide excellent mechanical and electrical properties but also can be disintegrated and bioresorbed *in vivo*. In addition, these characteristics can be regulated by covalent cross-linking between graphene and silk. GO is an ideal candidate for use as a 2D building block for constructing artificial nacre nanocomposites through interfacial interaction design due to its abundant functional groups, such as hydroxyl and carboxyl groups. Silk, as a natural protein with a hydrophobic antiparallel β sheet domain, also has functional groups such as amino and carboxyl groups. It is possible to design a dense network of synergistic interfacial interactions between GO flakes and silk protein molecules, including hydrogen bonding, hydrophobic interactions and amide covalent bonds. Hu et al. reported the high mechanical and conductive performance of graphene-based nanocomposites with silk through a layer-by-layer assembly approach and a vacuum filtration system (Hu et al., 2013a, Hu et al., 2013b). The ultimate tensile strength reached 300 MPa, and the conductivity was as high as 1,350 Sm^−1^. Their experimental results are better than our data, which may be caused by the different reduction methods. In our work, to avoid the toxic chemicals such as sodium borohydride (Gao et al., 2009), hydrazine (Kim et al., 2009), and hydriodic acid used in harsh conventional methods (Pei et al., 2010, Moon et al., 2010), we used a safe, nontoxic VC to reduce the nanocomposites. The reason for the difference between our results and previous results might be the presence of decomposition products (oxalic and guluronic acids) produced by VC during the reduction process (Zhang et al., 2010, Fernández-Merino et al., 2010). These products could form hydrogen bonds with residual oxygen groups on the graphene surfaces, thereby weakening the π-π interactions between the graphene sheets (Zhang et al., 2010) and affecting the overall mechanical and electrical conductivity of the films. However, compared with pure graphene films, the rGO/silk nanocomposite films have higher mechanical properties and maintain good electrical conductivity despite the use of VC to reduction. Furthermore, owing to the denser and more ordered interlayer structure (higher degree of crystallinity; Figure 2E), the covalently cross-linked films (C-rGO/silk) enhance the above characteristics. High mechanical strength and electrical conductivity of the films can maintain their structural and functional stability *in vivo*, which will be beneficial for applications in tissue engineering scaffolds and implantable electronic devices (Ryan et al., 2018).

The pure rGO film undergoes edge bending in the early stages of implantation *in vivo*. The rGO/silk and C-rGO/silk nanocomposite films maintained a flat shape owing to their high mechanical strength. However, in the late stages of film implantation, we found that all the films exhibited a deformation process from the edge to the bulk of the material. Furthermore, the local structures of the different films at the deformation/bending regions also show significant differences. The shape of the bending region of the pure rGO film is sharp, whereas the corresponding regions of the nanocomposite films are relatively smooth. Furthermore, a break occurred in a portion of the bending region of the non-cross-linked rGO/silk film group. To explain the phenomena that appear in these films during the bending process, we used FE analysis to simulate their dynamic processes. From our simulation results, the following characteristics can be found: (1) The non-cross-linked and cross-linked nanocomposite films can effectively resist deformation in the early stage, (2) the brick-and-mortar structure films undergo internal delamination in this process, (3) the non-cross-linked nanocomposite film is prone to fracture during bending, and (4) the occurrence of these bending processes proves that the films are subjected to continuous external force. From the tissue section results, we believe that this external force is derived from the contractile force induced by fibroblasts and myofibroblasts on implanted biomaterials during the FBR.

The FBR caused by biomaterial implantation is mainly divided into the following four steps. First, proteins such as immunoglobulin from the local environment are nonspecifically adsorbed on the surface of the materials in the initial stage of implantation; second, host inflammatory cells such as neutrophils and macrophages recognize the nonspecific protein-labeled materials as foreign objects, aggregate on the surface of the materials, and attempt to phagocytose and degrade them; third, macrophages recruit and transdifferentiate the fibroblasts and myofibroblasts around the materials; and finally, the formation of fibrotic collagenous capsules, granulation tissue (carrying abundant blood vessels), and FBGCs indicates that the FBR processes enter an end stage (Anderson et al., 2008, Sridharan et al., 2015). At present, most implanted biomaterials and devices can cause FBRs. Although the graphene family consists of bioinert carbon-based materials, graphene and graphene derivatives can still induce a certain degree of fibrous capsule encapsulation and the formation of FBGCs (Sydlik et al., 2015). According to our results, we found that macrophages aggregate at the bends in the films, followed by many myofibroblasts. Myofibroblasts are mainly distributed in damaged tissue sites and contracted wounds. They have a wide range of sources, mainly including fibroblast transition, mesenchymal stem cell differentiation, and macrophage transdifferentiation (Duffield et al., 2013, Hinz, 2010). Macrophages have an important influence on the aggregation and distribution of myofibroblasts. They can regulate and recruit myofibroblasts through the secretion of inflammatory factors, chemokines, and growth factors (Wynn and Vannella, 2016). We believe that, after implantation, the films form local deformation and depression regions owing to instability of the interface between the tissue and materials. These depressions become enriched in blood, body fluids, and proteins, thus causing the aggregation of more host cells, including macrophages. Eventually, these macrophages induce the formation of myofibroblast populations. Owing to the contractile force of myofibroblasts (Castella et al., 2010), the uneven distribution of these cells leads to uneven force on the films, which in turn causes film bending.

Why the rGO/silk and C-rGO/silk nanocomposite films with high mechanical strength are rapidly disintegrated in the late stage of implantation still needs further discussion. Most of the cells involved in the innate immune process can secrete different kinds of enzymes, which can play a key role in eliminating external microorganisms and degrading foreign materials. For example, immune cells such as macrophages, neutrophils, and FBGCs that are involved in the process of the FBR can secrete collagenase, matrix metalloproteinase, and myeloperoxidase (Al-Maawi et al., 2017, Sutherland et al., 1993). In our results, owing to the dense hydrophobic π-π bonds between the layers of graphene flakes, pure rGO films did not readily undergo enzymatic degradation *in vitro*, and only surface corrosion occurred. However, we found that many microcracks appeared on the surface of the rGO/silk and C-rGO/silk films under the combined action of peroxidase and protease, which may be the key factor in their rapid disintegration in the late stage of implantation *in vivo*. As early as 1991, Zhao et al. found that phagocytic cells can induce microcrack initiation in bioinert polymers *in vivo* (Zhao et al., 1991). At 10 weeks after implantation of a poly(ether urethane urea) film, the authors removed the FBGCs from the surface, and the exposed cell “footprints” showed surface cracks with depths and widths of several micrometers. There were no microcracks in adjacent areas where no cells were attached. These “footprints” (microcracks) are closely related to the peroxidase enzymes secreted by phagocytic cells. Interestingly, neither protease nor hMPO can effectively increase the number surface cracks, but they have synergistic effects, which may be related to the components (a large amount of an oxidatively decomposable inorganic component and a small amount of a proteolytically degradable organic component) of the nanocomposite films and the characteristics of these enzymes (surface charge and adsorption capacity). This phenomenon still needs to be studied in the future.

**Figure 8:**
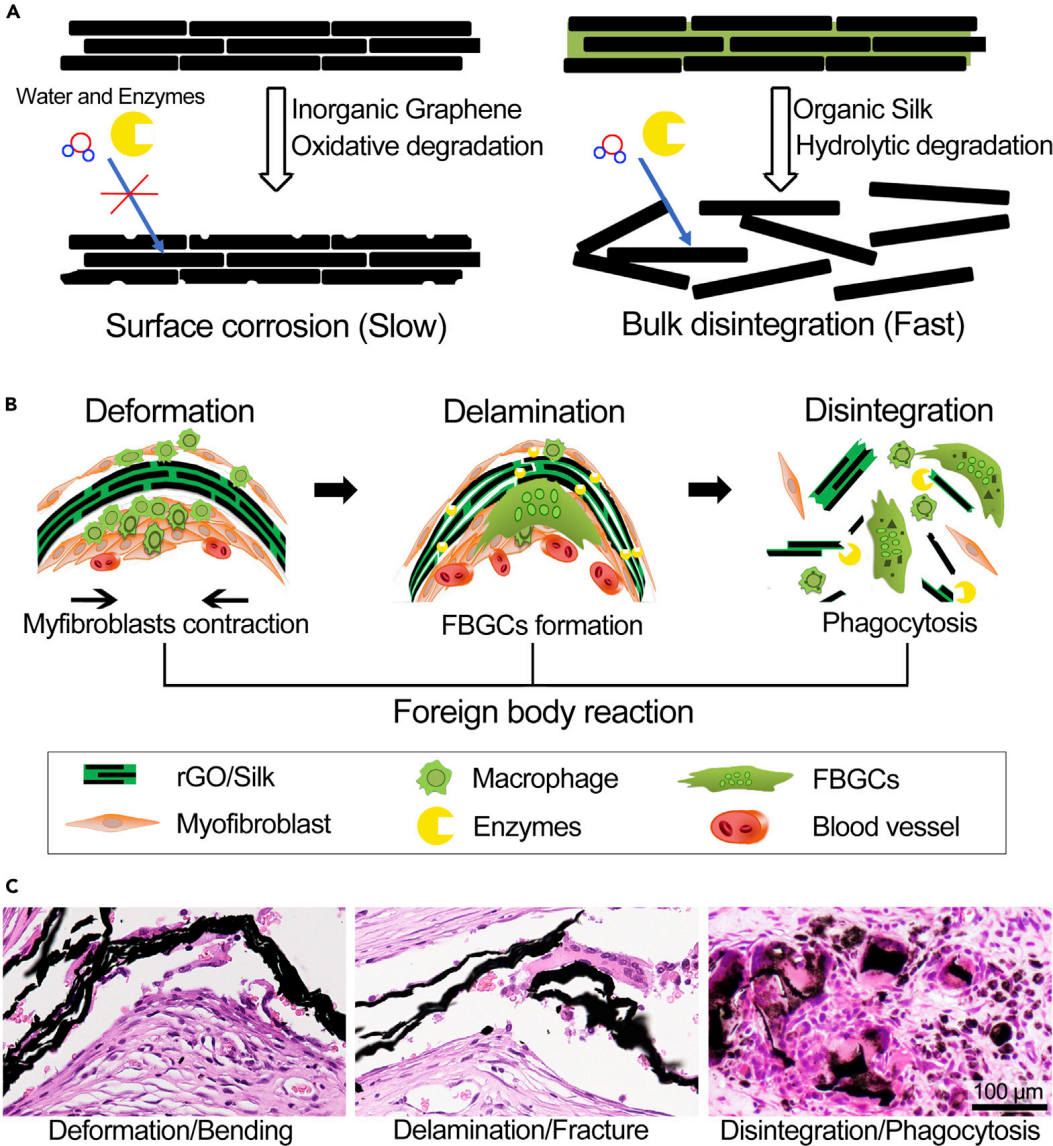
Summary Diagram of the Hypothesis-Driven Material Design and *In Vivo* Disintegration and Bioresorption of a Nacre-Inspired Graphene/Silk Films Caused by the FBR (A) The basic mechanism of the slow degradation of a macroscopic graphene film (surface erosion) and fast degradation of a nacre-inspired graphene-based nanocomposite film (bulk disintegration). (B) Schematic illustration of the deformation, delamination, and disintegration processes of nanocomposite film induced by the FBR. (C) Representative images of the H&E staining of histological sections of rGO/silk films at different stages. The images show the *in vivo* disintegration and bioresorption processes of the nacre-inspired nanocomposite film.

One of the classic *in vivo* degradation modes of biodegradable polymers is the construction of biodegradable “bridging” bonds between nondegradable polymer monomers (Gombotz and Pettit, 1995). After these “bridging” bonds are enzymatically hydrolyzed *in vivo*, the polymers disintegrate/decompose into monomers and eventually are metabolized. We chose silk as the “bridging” protein of this nacre biomimetic system, which is mainly due to its excellent mechanical retention, biodegradation, and synergistic interactions with graphene compared with those of other natural proteins such as collagen and gelatin, especially in the case of nanocomposites containing a small proportion of protein (Li et al., 2014, Qian et al., 2018). The original intention of our design is for the bioinspired nacre structure to not only maintain the structural stability in the early stage but also function as the organic “mortar” as a “bridging” protein that can be degraded *in vivo* (Figure 8A). From our results, it was found that the nanocomposite films showed deformation, delamination, and disintegration at the late stage of implantation (3 months) and produced a large amount of dispersed debris, especially the non-cross-linked rGO/silk films. We also found that macrophages and FBGCs phagocytose debris. FBGCs, which consist of numerous fused macrophages, can secrete a large amount of strong oxidases and proteases and have phagocytic ability (Wang et al., 2018). In the later stage of the FBR, the production of more debris by the nanocomposite may induce the formation of more FBGCs, which ultimately accelerate the disintegration and phagocytic degradation of the film (Figures 8B and 8C). Although the overall volume of the material decreased significantly during this process, no metabolically accumulated graphene debris was found in the main organs (Figure 7D).

In summary, the artificial nacre graphene/silk nanocomposites we have developed not only maintain good mechanical stability and electrical conductivity but also impart their disintegration ability *in vivo* to the bioinert macroscopic graphene film. The addition of a small amount of silk protein to the nanocomposite system effectively improved the overall mechanical properties of the graphene-based films, maintained their high electrical conductivity, and reduced the FBR *in vivo*. Importantly, the films underwent deformation, delamination, disintegration, and phagocytosis at the later stage of implantation. These behaviors are mainly attributed to the deformation of the films caused by the contraction of myofibroblasts, the enzymatic degradation of the “bridging” silk protein, and the phagocytic ability of phagocytic cells. Biological factors may be involved in the whole disintegration process from the macroscopic to the microscopic scale. Such *in vivo* disintegrable graphene-based films based on nacre-biomimetic structures have great potential applications in degradable bioelectrodes, implantable devices, and tissue engineering scaffolds in the future.

### Limitations of the Study

In this study, we have demonstrated that the distribution and number of macrophages and myofibroblasts change at the early and late stages of implantation *in vivo* (Figure 5). However, it is difficult to track the migration and differentiation behavior of the immune cells, which requires specific transgenic mice and time-lapse microscopy for real-time observation. In future work, we will further analyze the spatiotemporal dynamic behavior of immune cells involved in foreign-body reaction, including migration, proliferation, and differentiation. We also know that there are complex biological mechanisms in the process of material degradation and foreign-body reaction and a variety of immune cells are involved in this process. It will be interesting to explore that cellular autophagy and ubiquitination pathways are involved in this process. We will further analyze the signal pathways of cell phagocytosis in the process of foreign-body reaction in our future work.

## Methods

All methods can be found in the accompanying Transparent Methods supplemental file.

## Data and Code Availability

All relevant data are available from the authors upon request.

## Supplemental Information

Document S1. Transparent Methods and Figures S1–S7
