# Supplementary material for: In Vivo Disintegration and Bioresorption of a Nacre-Inspired Graphene-Silk Film Caused by the Foreign-Body Reaction
Source: iScience. 2020 May 13;23(6):101155. doi: 10.1016/j.isci.2020.101155 (PMC7251954; doi:10.1016/j.isci.2020.101155)
Supplement: Document S1. Transparent Methods and Figures S1–S7 [file mmc1.pdf]

iScience, Volume *23*

Supplemental Information

In Vivo Disintegration and Bioresorption of a Nacre-Inspired Graphene-Silk Film Caused by the Foreign-Body Reaction

Linhao Li, Yanbing Liang, Guohang Wang, Peng Xu, Lingbing Yang, Sen Hou, Jin Zhou, Lizhen Wang, Xiaoming Li, Li Yang, and Yubo Fan

Supplemental Information

In vivo disintegration and bioresorption of a nacre-inspired graphene-silk film caused by the foreign-body reaction

Linhao Li, Yanbing Liang, Guohang Wang, Peng Xu, Lingbing Yang, Sen Hou, Jin Zhou, Lizhen Wang, Xiaoming Li, Li Yang, and Yubo Fan

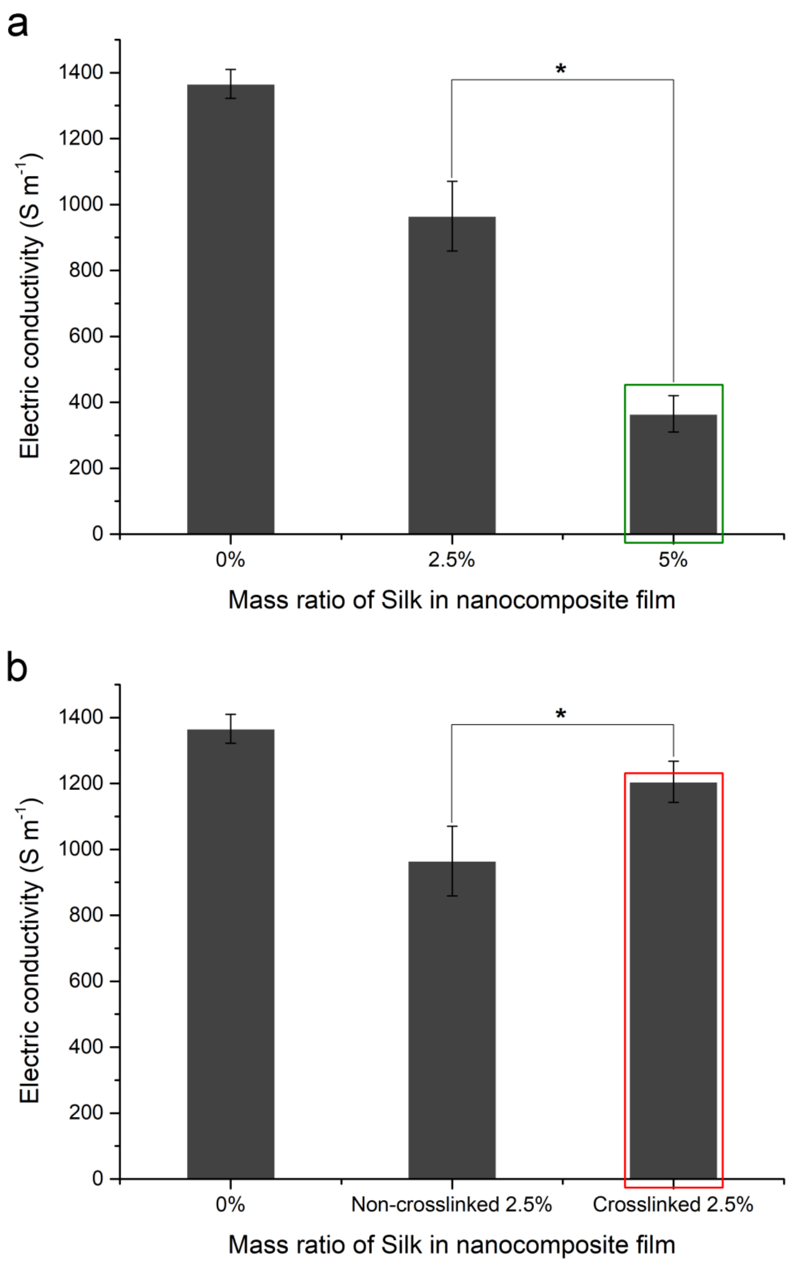

Figure S1. Electrical conductivity of different mass ratio of silk in the nanocomposite films. Related to Figure 1e. (a) Electrical conductivity of the nanocomposite films containing 0%, 2.5% and 5% silk. The electrical conductivity of the nanocomposite film containing 5% silk showed a significant decrease, which was about 3 times lower than that of the pure rGO film. $^*P < 0.05$. Data are presented as the mean ± SD (n=4). **(b)** Electrical conductivity of the nanocomposite films containing 0%, noncrosslinked 2.5%, and crosslinked 2.5% silk. $^*P < 0.05$. Data are presented as the mean ± SD (n=4).

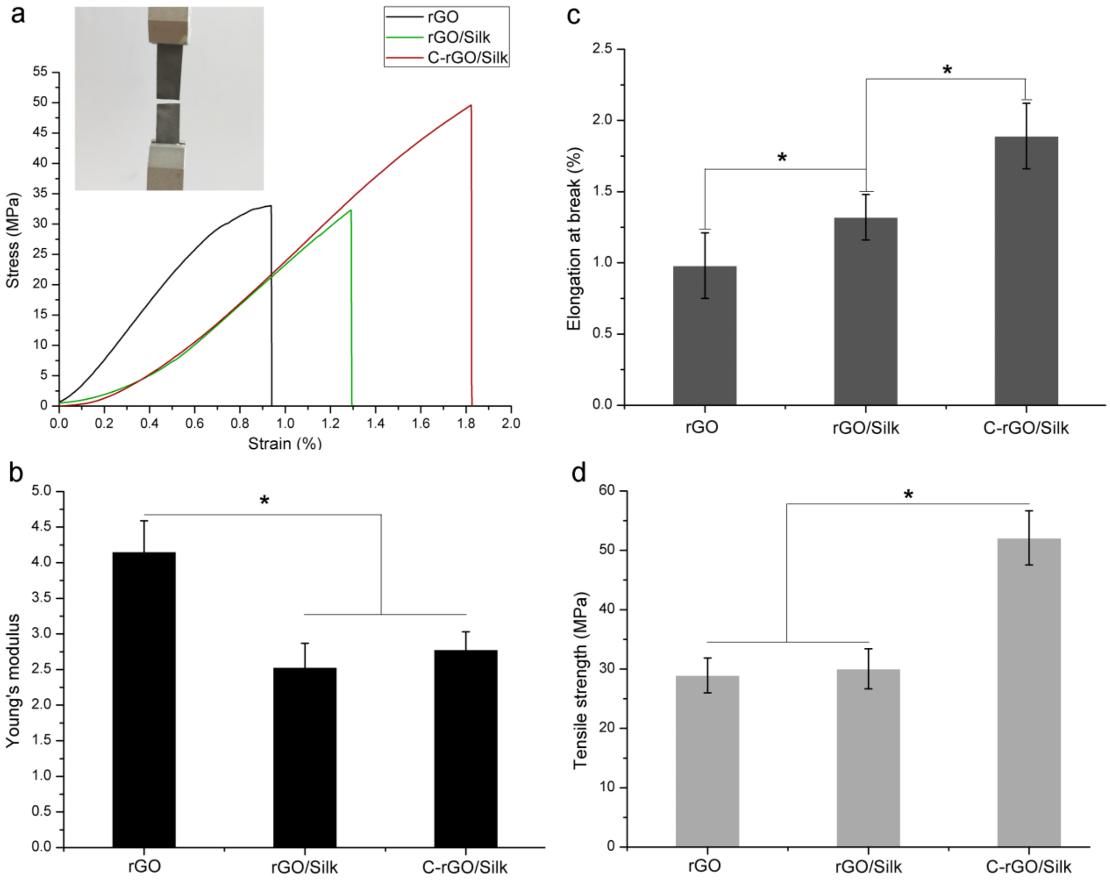

Figure S2. Mechanical properties of different films. Related to Figure 1f. (a) The representative stress-strain curve of different films. **(b, c and d)** Young's modulus, elongation at break and tensile strength of the different films. *P < 0.05. Data are presented as the mean ± SD (n=3).

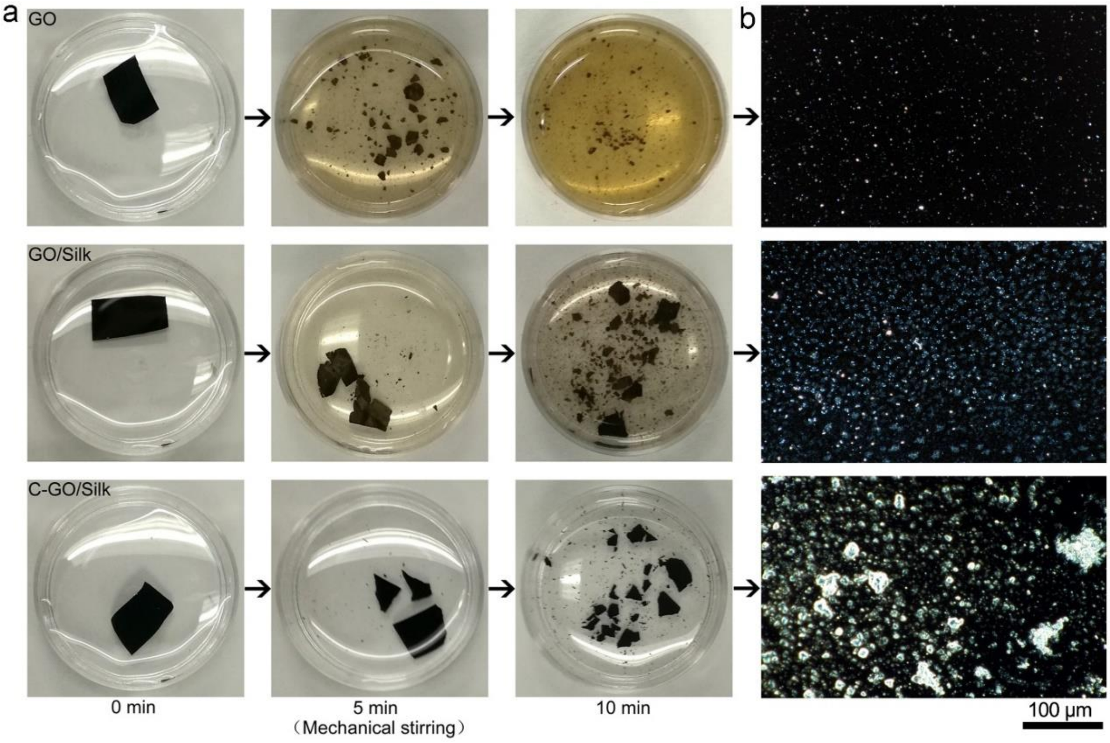

Figure S3. Analysis of the interactions between graphene oxide and silk with and without crosslinking. Adding silk and crosslinking enhances the structural retention of GO films. Related to Figure 2b. (a) Photographs of GO, GO/silk, C-GO/silk films after being soaked and stirred in water for 0, 5, and 10 minutes with a magnetic stirrer at the same rotational speed. **(b)** Polarization microscopy images of dispersed solutions (suspensions) of different films stirred by magnetic force for 10 minutes (× 400).

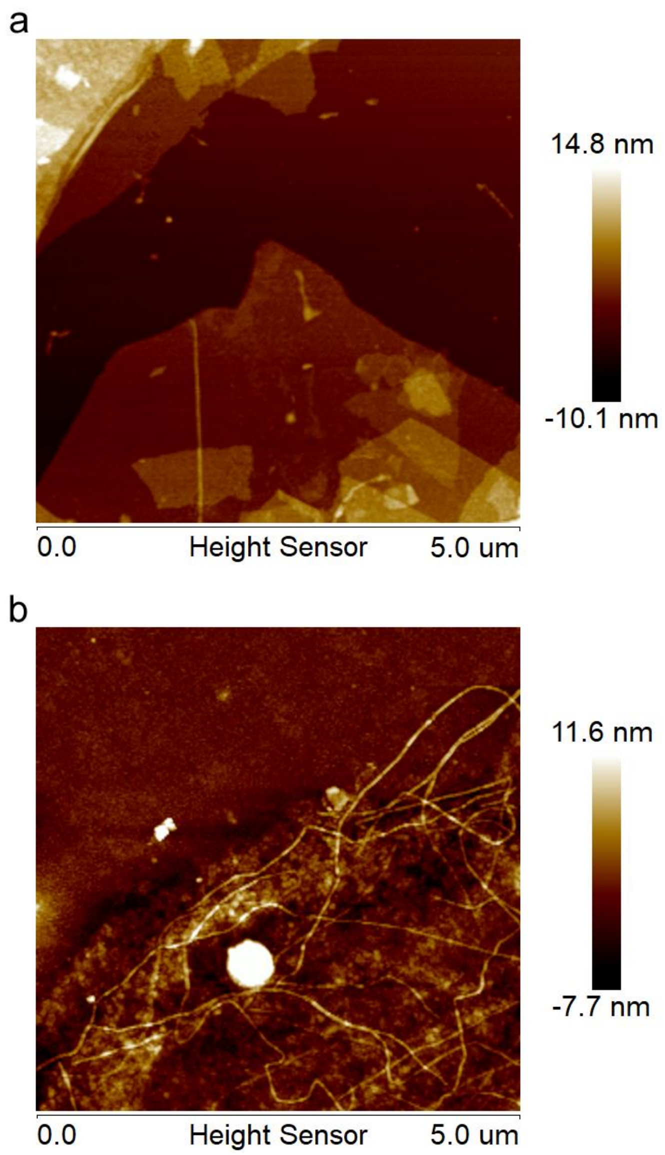

Figure S4. Crosslinking promotes the adhesion of silk nanofilaments on the surface of GO flakes. Related to Figure 2c. (a and b) AFM images of pure GO flakes and dispersed solutions (suspensions) of C-GO/silk nanocomposites stirred by magnetic force for 10 minutes. The surface of part GO flakes became rough and nanofilaments appeared.

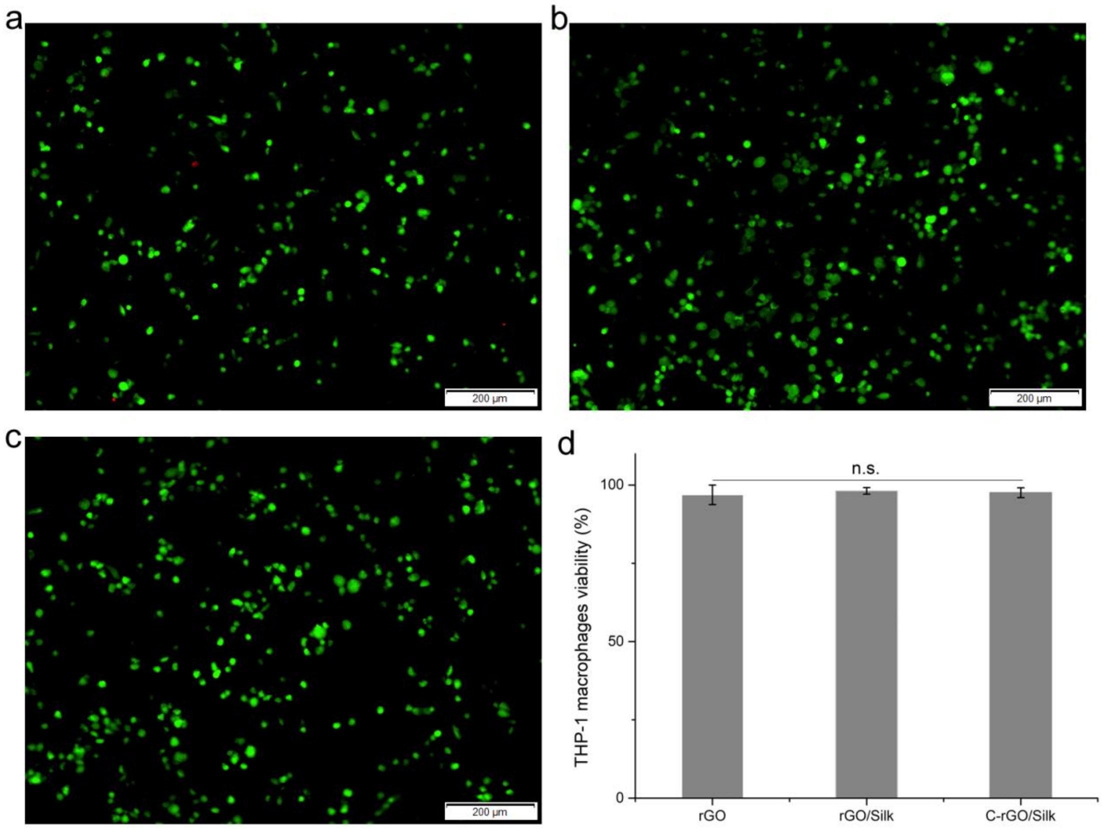

Figure S5. Cytotoxicity assay for macrophages cultured on different films. Related to Figure 3a. (a, b, and c) Fluorescence images of live/dead macrophages cultured on rGO, rGO/silk, and C-rGO/silk films. Calcein-AM (green) indicates live cells while PI (red) indicates dead cells. **(d)** Cell viability rates in different films. Data are presented as the mean ± SD (n=3). "n.s." means no significance.

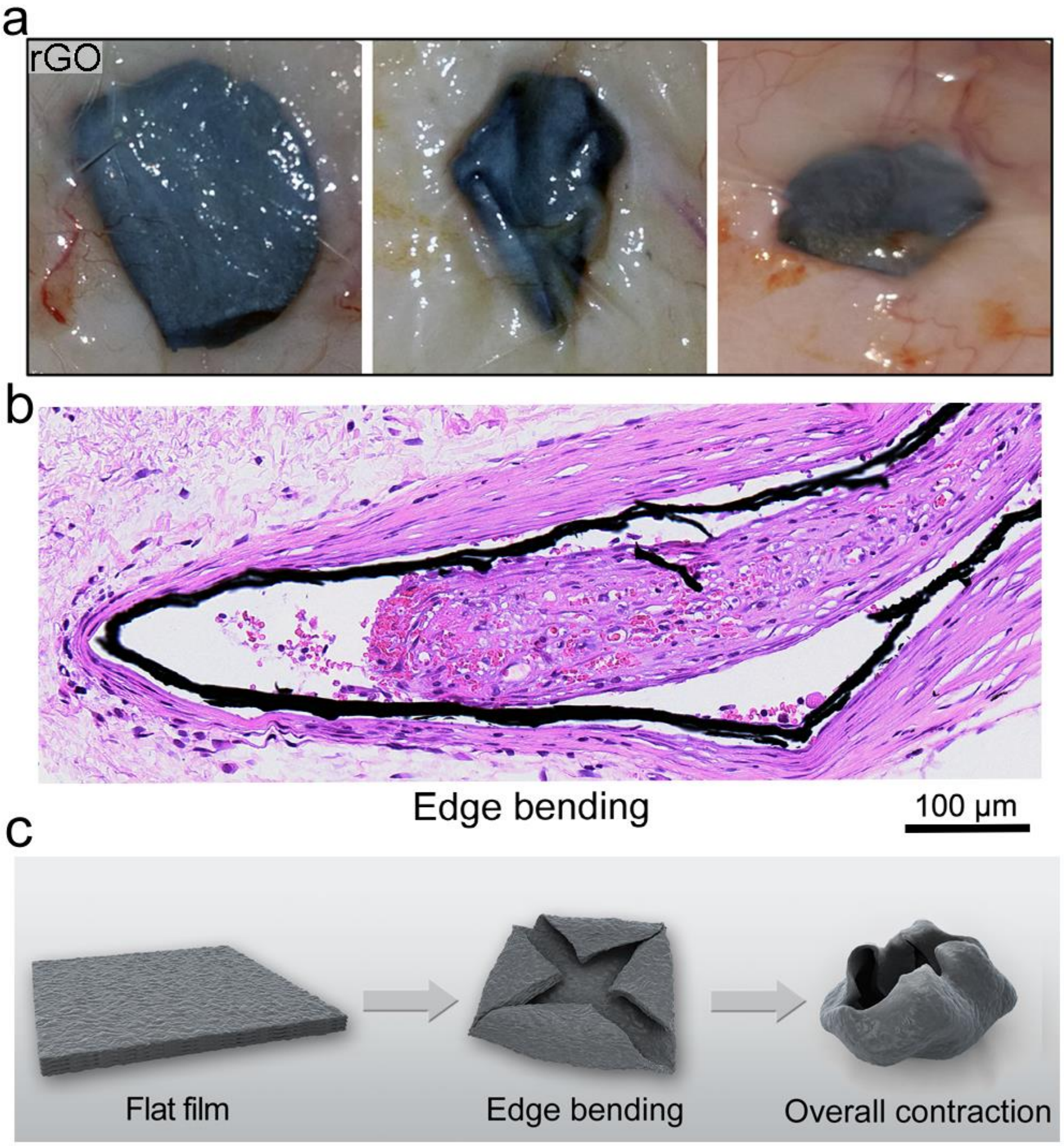

Figure S6. The deformation process of rGO film *in vivo*. Related to Figure 3e. (a) Photographs of rGO/tissue *in situ* after implantation for 4-, 8- and 12-weeks. **(b)** Representative image of the H&E staining of histological sections of rGO film after 4-weeks of implantation, show the numerous host cells appeared at the bend of the edge (inside of the film). **(c)** Schematic illustration of the process of deformation of a flat rGO film *in vivo*.

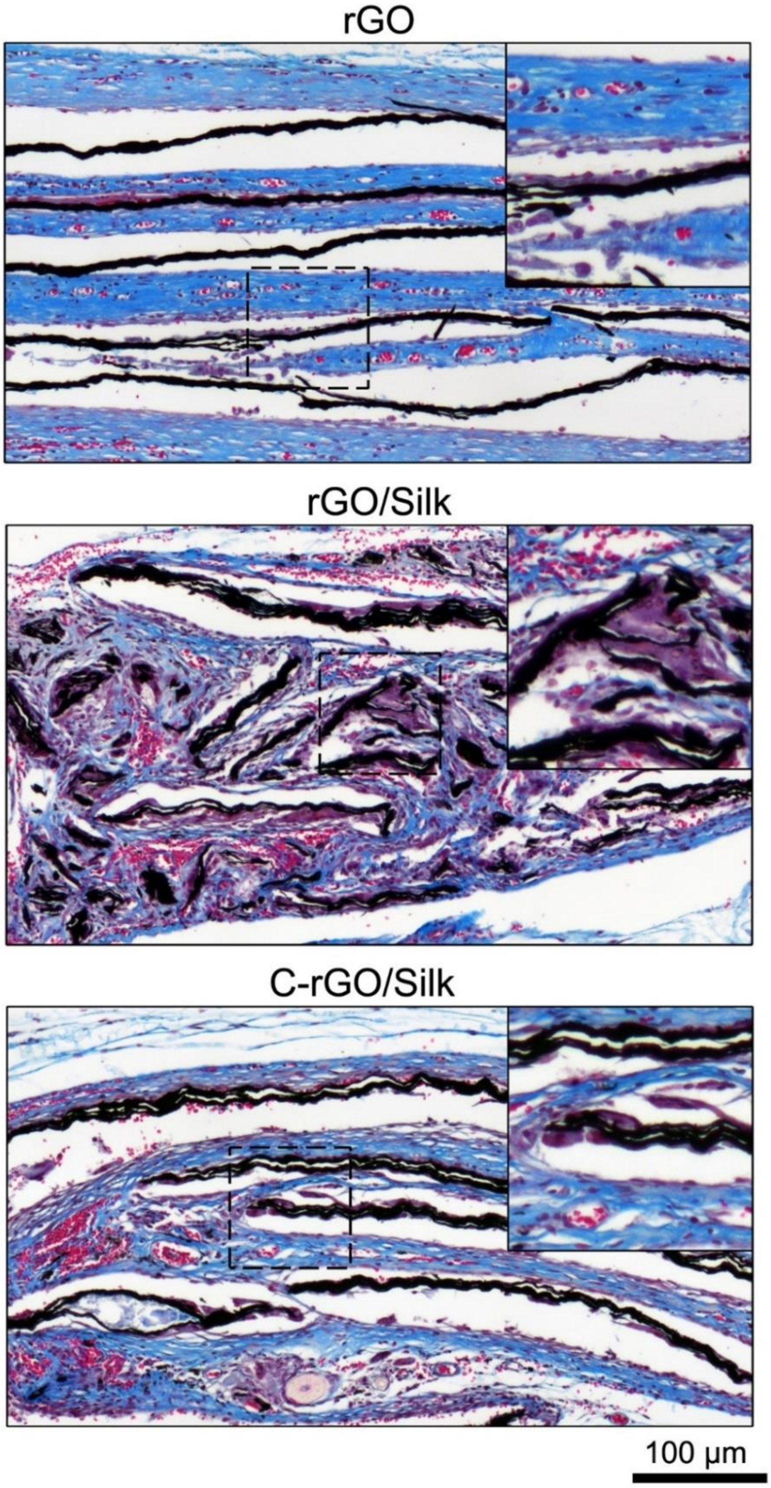

Figure S7. Foreign-body reaction to different films at the late stage of implantation. Related to Figure 4 and Figure 7. Representative images of the Masson's trichrome staining of histological sections of rGO, rGO/silk, and C-rGO/silk films at 12 weeks after implantation, show the different films have different *in vivo* disintegrated behaviors and collagen capsule formation.

Transparent Methods

Fabrication of graphene/silk nanocomposite films

Graphene oxide (GO) solution (few-layer, diameter > 500 nm, 2 mg/L) was purchased from Nanjing XFNano Material Tech Co., Ltd. (China). To extract silk fibroin (SF) protein, cocoons from *B. mori* (ZheJiang XingYue Biotechnology Co., Ltd., China) were boiled twice in an aqueous solution of 0.02 M Na_2CO_3 (Sigma-Aldrich) for 1 h, rinsed 5 times with ultrapure water (Millipore) and dissolved in 9.3 M LiBr (Sigma-Aldrich) at 60 °C to generate a 10% (w/v) solution. The solution was dialyzed (Mw = 3500 Da, Pierce Chemical, Co. USA) against ultrapure water for 3 days, and the water was changed daily to remove ions and other impurities. The solution was collected, filtered and stored at 4 °C. SF solution (pH = 7) was dropped into the GO solution under vigorous stirring to give a final mixture (10 mL) with a GO concentration of 0.2% (w/v) and GO/SF solid mass ratios of 95:5 and 97.5:2.5. The pH of the solution was adjusted to 10 with 1 M NaOH. Afterward, the GO/silk nanocomposites were fabricated by vacuum filtration of the suspensions prepared above using a vacuum filtration apparatus and nylon filtration membranes (pore size of 0.2 μm, Sigma-Aldrich). Film thickness was controlled by the volume of solution being filtered. To crosslink the GO/silk films, 2 mM 1-ethyl-3-carbodiimide hydrochloride (EDC, ThermoFisher, Co. USA) and 5 mM N-hydroxysuccinimide (NHS, ThermoFisher, Co. USA) were added to 0.1 M 2-(morpholino)ethanesulfonic acid (MES, ThermoFisher, Co. USA) buffer (pH = 5.6) as a reaction solution and reacted overnight at room temperature. Finally, for VC reduction, the nanocomposite films were immersed in an aqueous solution of VC (30 g/L) at 70 °C

for 6 h.

Structure and characterization of the nanocomposite films

The morphology of rGO, rGO/silk, and C-rGO/silk films was characterized by field-emission SEM (S-4800, Hitachi, Japan) with an accelerating voltage of 5 kV. Optical polarization images of GO, GO/silk, and C-GO/silk films were obtained using an industrial microscope (LV150N, Nikon, Japan).

Electrical conductivity measurements were performed by using an HMS-3000 Hall measurement system with four-point probes. Current-voltage curves were obtained from 1 × 1 cm^2 films to extract the conductivity.

Atomic force microscopy (AFM) was performed by using a Nanoscope VIII (Bruker, USA) in intermittent tapping mode at a scan rate of 1 Hz by using aluminum silicon cantilevers with a typical tip radius below 10 nm (Bruker, USA). A 20 μL aliquot of each solution was deposited onto freshly cleaved mica and incubated for 2 h. Then, the samples were rinsed with ultrapure water and gently dried in air.

The mechanical properties of the different scaffolds were determined using a tabletop uniaxial testing instrument (AG-IS, Shimadzu, Japan) with a 50 N load cell under a strain ramp rate of 1 mm/min and ambient conditions (RH ~70%). All the films were cut into a rectangular shape with a size of 1 × 2 cm^2. The thickness range of the films used in the mechanical testing was approximately 5.0 ± 2.0 μm. The tensile strength, Young's modulus, and elongation at break were obtained from the stress-strain curves.

The water contact angles of the different films were measured using a Model 200 video-based optical system (JY-82C, Chengde DingSheng Co., China). An initial distilled water volume of 5 μL was used in each measurement after 1 s of exposure to ambient temperature.

Chemical and structural analyses of the SF, GO, GO/silk, and C-GO/silk films were performed by Fourier transform infrared (FTIR) spectroscopy over a range of 4000-400 cm^{-1}. The FTIR spectra of different samples were obtained by a Nicolet spectrometer system (System 2000, PerkinElmer) with a deuterated triglycine sulfate (DTGS) KBr detector. XRD patterns were acquired on a Rigaku D/MAX-2500 using CuKα radiation and a scanning speed of 4°/min.

For finite element (FE) simulation analysis, a 2D FE model is developed using the commercial software ABAQUS v6.13. In the simulation, a brick-and-mortar structural unit (3 μm ×500 nm) is used. The nacre structure in the FE model comprises a staggered arrangement of oriented thin graphene bricks bonded by an SF biopolymer layer, which is modeled as a cohesive zone with traction separation. The isotropic elastic modulus of the brick (graphene) is 100 GPa. The SF modulus is 5 GPa, and the failure strength is 80 MPa of plastic deformation before failure. Three FE models with different cohesive modes are built: a film composed of only rGO; a film composed of noncrosslinked graphene and SF (rGO/silk); and a film composed of crosslinked graphene and SF (C-rGO/silk). We set 5 MPa shear stress along the surface of the film from the outside to the inside to bend the film. The interfacial adhesion strengths of the rGO model, rGO/silk model, and C-rGO/silk model are 30 MPa, 20 MPa and 50 MPa,

respectively. The simulated film bending process is mainly for reproducing the deformation and delamination of the nanocomposite films.

Cell viability

Human THP-1 macrophages were cultured in RPMI 1640 medium (Gibco, USA) supplemented with 20% fetal bovine serum (FBS, Gibco, USA). The films were immersed in 70% ethanol for 30 minutes, dried under sterile conditions and exposed to UV radiation for 1 h; then, the films were washed 3 times with PBS for 5 minutes each and incubated with serum-free 1640 medium for 24 h before cell seeding. The cells were seeded onto the films in a 24-well plate at a density of 1×10^5 cells/well and cultured with RPMI 1640 medium and 20% FBS at 37 °C, 5% CO_2 and 95% humidity. The cytotoxicity of the nanofibers was determined using a Live-Dead cell staining kit (Invitrogen, USA). Live cells were stained with only calcein-AM (green). Dead cells were stained with the non-cell-permeable dye PI (red).

Subcutaneous implantation

The films were cut into a square shape with dimensions of 1 cm × 1 cm × 500 μm (L×W×T) under sterile conditions. Sprague-Dawley rats (male, body weight of 220-230 g) were selected for *in vivo* implantation. The use of rats conformed to the Guiding Principles for the Care and Use of Animals of our institute and was approved by the Animal Care and Use Committee of our institute. Four small midline incisions were made on the dorsum of each rat, and the films were introduced in lateral subcutaneous pockets created by blunt dissection. After 14, 28, 56, and 84 days, the rats were

sacrificed, and the implanted films and major organs were retrieved.

Histology and immunofluorescence staining

The implanted films along with the surrounding tissues and major organs were harvested at different time points and fixed in 4% polyformaldehyde overnight at 4 °C. Samples were dehydrated through a series of graded alcohol baths and in xylene, embedded in paraffin and sliced into 5 μm thick sections. These sections were subjected to H&E staining and Masson's trichrome staining to assess the foreign-body reactions (FBRs) and graphene debris residue in major organs. Immunofluorescence staining was performed using monoclonal anti-CD68 and anti-α-SMA antibodies for rat macrophages and myofibroblasts. All sections were evaluated with a fluorescence and brightfield slide scanner (Leica SCN400 F, Wetzlar, Germany). Image analysis was performed using ImageJ software to quantify both CD68 and α-SMA expression at different time points.

Enzymatic treatment

Before treatment, different nanocomposite films were cut to a size of 5×5 mm^2 and immersed in PBS (0.01 M, pH = 7.4) for 0.5 h. For protease treatment, each film was placed in a 1.5 mL centrifuge tube, and 1 mL of a 0.5 mg/mL protease XIV (Sigma) solution was added and incubated at 37 °C for 3 days with constant shaking. The protease XIV solution was changed every 12 h to ensure the activity of the enzyme. For peroxidase treatment, each film was placed in a 200 μL centrifuge tube, and 100 μL of 0.1 mg/mL hMPO (180 U, Abcam) solution was added to the centrifuge tube and

incubated at 37 °C for 24 h. Meanwhile, 2.5 μL of 10 Mm H_2O_2 was added to each solution to trigger the degradation reaction. Due to the consumption of H_2O_2, it is necessary to refill the tube with 2.5 μL of fresh 10 mM H_2O_2 solution every 5 h. For the double enzyme treatment, the protease and peroxidase treatments were still carried out according to the above procedures in sequential order. Finally, the enzymatically treated films were washed 3 times with PBS buffer, and the surface morphology was observed by polarization microscopy and SEM.

Statistical analysis

The results are expressed as the means ± standard deviations. Statistical analysis was performed using Student's t-test as well as one-way analysis of variance (ANOVA) followed by the Tukey HSD test for post hoc comparison (Origin 9, OriginLab, Northampton, MA). Differences were considered statistically significant if $p < 0.05$.
